# Clinical and histopathological analyses of VEGF receptors peptide vaccine in patients with primary glioblastoma - a case series

**DOI:** 10.1186/s12885-020-6589-x

**Published:** 2020-03-12

**Authors:** Ryota Tamura, Yukina Morimoto, Kenzo Kosugi, Mizuto Sato, Yumiko Oishi, Ryo Ueda, Ryogo Kikuchi, Hideaki Nagashima, Shinobu Noji, Yutaka Kawakami, Hikaru Sasaki, Kazunari Yoshida, Masahiro Toda

**Affiliations:** 1 Department of Neurosurgery, Keio University School of Medicine, 35 Shinanomachi, Shinjuku-ku, Tokyo, 160-8582 Japan; 2 Department of Neurosurgery, Hiratsuka City Hospital, Hiratsuka, Kanagawa 254-0019 Japan; 3 Division of Cellular Signaling Institute for Advanced Medical Research, Keio University School of Medicine, 35 Shinanomachi, Shinjuku-ku, Tokyo, 160-8582 Japan

**Keywords:** VEGFR, Peptide vaccine, Glioblastoma, Bevacizumab

## Abstract

**Background:**

The expression of vascular endothelial growth factor (VEGF)-A/ VAGF receptors (VEGFRs) signaling plays a pivotal role in the tumor angiogenesis and the development of the immunosuppressive tumor microenvironment in glioblastomas. We have previously conducted exploratory clinical studies investigating VEGFRs peptide vaccination with and without multiple glioma oncoantigens in patients with recurrent high-grade gliomas. Recently, an exploratory clinical investigation of VEGFRs peptide vaccination was conducted in patients with progressive neurofibromatosis type 2. Those studies suggested that cytotoxic T lymphocytes (CTLs) induced by the vaccination can directly kill a wide variety of cells associated with tumor growth, including tumor vessels, tumor cells, and immunosuppressive cells expressing VEGFR1 and/or 2. In the present study, synergistic activity of the combination of VEGFRs peptide vaccination with chemotherapy was evaluated.

**Methods:**

We performed the first clinical trial to assess VEGFR1 and 2 vaccination along with temozolomide (TMZ) -based chemoradiotherapy for the patients with primary glioblastomas. Furthermore, histopathological changes after the vaccination were evaluated using paired pre- and post- vaccination specimens.

**Results:**

The disappearance of radiographically enhanced lesion was observed in 2 patients after the vaccination, including one in which the methylation of the O6-methylguanine-DNA methyltransferase (MGMT) promoter was not observed. The histopathological findings of pre- and post-vaccination specimens demonstrated that tumor vessels showed negative or slight VEGFRs expressions after the vaccination and most endothelial cells were covered with PDGFR-β-positive pericytes. Notably, CTLs induced by VEGFRs peptide vaccination attacked not only tumor vessels but also tumor cells and regulatory T cells expressing VEGFRs even in recurrent tumors.

**Conclusions:**

VEGFR1 and 2 vaccination may have a preliminary synergistic effect when administered with TMZ. The limitation of the present study was the paucity of the number of the samples. Further studies involving more patients are warranted to confirm the findings of this study.

**Trial registration:**

This study was registered as UMIN000013381 (University Hospital Medical Information Network-Clinical Trial Registry: UMIN-CTR) on 5 March, 2014 and with the Japan Registry of Clinical Trials (jRCT) as jRCTs031180170 on 1 March, 2019.

## Background

Angiogenic factors are important for the growth of malignant tumors, and vascular endothelial growth factor (VEGF)-A/ VEGF receptors (VEGFRs) signaling is the most potent [1]. Glioblastoma is a highly malignant tumor that exhibits extensive vascularity. The expression of VEGF-A/ VEGFRs is strongly upregulated in glioblastoma, and the expression degree correlates with the grade of malignancy and prognosis in malignant glioma [2, 3]. It has been reported that both VEGFR1 and VEGFR2 are expressed on not only vascular endothelial cells, but also tumor cells [4]. VEGFR1 signaling is critical for tumor growth [5]. VEGFR2 plays an important role in the proliferation of tumor stem cells [6]. Furthermore, VEGF/VEGFR signaling plays a pivotal role in the development of the immunosuppressive tumor microenvironment in glioblastomas [7].

Therefore, VEGF-A and VEGFRs targeted anti-angiogenic therapies have been previously used in glioblastomas. Bevacizumab, which targets circulating VEGF-A, and multikinase inhibitor, such as cediranib, sunitinib, and sorafenib, were associated with favorable event-free survival in patients with glioblastomas [8–12]. In addition, combinational therapy with anti-angiogenic therapy and chemotherapy has been an attractive treatment strategy in glioblastomas, because anti-angiogenic therapy induces a functional normalization of the tumor vasculature, increasing tumor cell exposure, and enhancing the activity of co-administered chemoradiotherapies [13]. The combinational therapy involving sorafenib plus daily temozolomide (TMZ), which is the primary chemotherapy used globally for glioblastoma treatment [14], was used for the recurrent glioblastomas, indicating that it is feasible, safe, and has some effect on patients [15].

Cancer immunotherapy has become the fourth preferred modality of cancer treatment after surgery and chemoradiotherapy. Peptide vaccination is an immunotherapy that aims to activate cytotoxic T lymphocytes (CTLs) in patients by inoculating antigen peptides. We have previously conducted exploratory clinical studies investigating VEGFRs peptide vaccination with and without multiple glioma oncoantigens in patients with recurrent high-grade gliomas [16, 17]. Recently, VEGFRs peptide vaccine was used in patients with progressive neurofibromatosis type 2 (NF2) [18]. In the study, the number of Foxp3-positive regulatory T cells (Tregs) decreased after the vaccination, suggesting that the CTLs induced by the vaccination can directly kill a wide variety of cells associated with tumor growth, including tumor vessels, tumor cells, and Tregs expressing VEGFR1 and/or VEGFR2. Furthermore, the combinational usage of chemotherapy and immunotherapy is also effective with synergistic activity [19], because chemotherapy suppressed immunosuppressive T cells and immunotherapy sustains the proliferation of potential effector immune cells [20, 21].

Based on these backgrounds, in this trial, VEGFR1 and 2 vaccine was used with TMZ-based chemoradiotherapy for the patients with primary glioblastomas [14]. VEGFRs peptide vaccination might have both the advantages of anti-angiogenic therapy and immunotherapy. In addition, we successfully evaluated the histopathological changes after the VEGFR1 and 2 vaccination using paired pre- and post-vaccination patient-derived specimens, proving the synergic effects when administered with chemotherapy.

## Methods

### Trial overview

The present study was an exploratory phase I and II clinical trial to assess the feasibility and effectiveness of VEGFR1 and 2 peptide vaccination in primary glioblastoma. All protocols were approved by the Keio University School of Medicine Ethics Committee (reference number: 20130461), and conducted in accordance with the Helsinki declaration on experimentation on human subjects. The trial was registered at UMIN (UMIN000023565) and jRCT (jRCTs031180170). The authors affirm that human research participants provided informed consent to participate in the study and for publication of their data.

### Patient eligibility

Patients with high-grade glioma (WHO grade III or IV) after standard treatment (surgical removal + radiotherapy (RT) concomitant with TMZ [14]) were enrolled in this clinical study at the Department of Neurosurgery, Keio University School of Medicine. Patients also had to show positive for the genomic DNA typing test for HLA-A*2402 (HLA Laboratory, Kyoto, Japan). Details of the inclusion and exclusion criteria are provided in Table 1.

**Table 1:** Inclusion and exclusion criteria

Inclusion criteria	Exclusion criteria
• Histological diagnosis of high-grade glioma (WHO grade III or IV) • Announcement of a diagnosis • Positive genomic DNA typing test for HLA-A*2402 (HLA Laboratory, Kyoto, Japan) • Age between 16 and 79 • Eastern Cooperative Oncology (ECOG) performance status (PS) 0–2 • Completion of standard treatment (surgical removal + radiotherapy concomitant with temozolomide) • No prior surgery, irradiation, or chemotherapy 4 weeks before entry to the study • No uncontrollable pleural, peritoneal or cardiac effusion • Life expectancy > 3 months • Written informed consents are obtained. Lab values prior to vaccine • Neutrophil count ≥1000/mm^3^ • Platelet count ≥500,00/mm^3^ • Hemoglobin level ≥ 8.0 g/dl, a • Aspartate aminotransferase and alanine aminotransferase ≤4.0x the institutional normal upper limits • Total bilirubin ≤1.5x • Creatinine ≤2.0 mg/dL • No uncontrollable pleural, peritoneal or cardiac effusion	• The presence of uncontrollable severe infectious diseases • Adverse event of National Cancer Institute - Common Toxicity Criteria (NCI-CTC) grade 3 or 4 • Unable to take anything orally over 24 h • Other uncontrolled malignant diseases • Myeloproliferative diseases • After allogeneic hematopoietic stem cell transplantation • Active autoimmune diseases • Severe drug allergy • Concurrent treatment with steroids or immunosuppressive agents • Pregnant women or patients who planned to become pregnant during the study period • Psychiatric disorders • Unhealed wound • Decision of unsuitability by the principal investigator or the physician in charge.

### Peptides

**Fig. 1:**
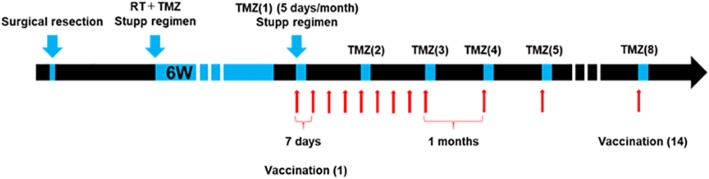
The Protocol for this clinical trial. The scheme of the Protocol for this clinical trial is shown. VEGFR1 and R2 peptide were injected eight times every week and then six times monthly (a total of 14 times). Vaccination was synchronized with adjuvant TMZ

Good manufacturing practice (GMP)-graded VEGFR1-A24–1084 peptide (SYGVLLWEIF) and VEGFR2-A24–169 peptide (RFVPDGNRI) were synthesized by BCN Peptides S.A. according to a standard solid-phase synthesis method and purified by reversed-phase high-performance liquid chromatography (HPLC). The purity (> 95%) and the identity of the peptides were determined by analytical HPLC and mass spectrometry, respectively. VEGFR1-A24–1084 and VEGFR2-A24–169 peptide (2 mg of each) were emulsified together with 1 ml of incomplete Freund’s adjuvant (Montanide ISA-51 VG, SEPPIC, Paris) and injected subcutaneously at infra-axillary and inguinal lymph nodes eight times every week and then six times monthly (a total of 14 times). Vaccination was synchronized with adjuvant TMZ [14] (Fig. 1). The period of this study was 12 months starting after the 1st vaccination.

### Outcomes and assessments

The primary endpoints were safety and clinical efficacy of the vaccination [median overall survival (OS) time]. OS was defined as the interval from the date of commencement of treatment to the date of death. The secondary endpoints were radiographical and immunological responses. Toxicities were assessed with the Common Terminology Criteria for Adverse Events version 4.0 (CTCAE ver4.0) at each visit. To evaluate the clinical response, magnetic resonance imaging (MRI) were performed within 2 weeks before the first cycle, and then after 8, 12 and 14 cycles (3, 6, and 12 months). Radiographical response was evaluated by Response Assessment in Neuro-Oncology (RANO) and immunotherapy RANO (iRANO) using gadolinium (Gd) -enhanced T1 weighted images and fluid attenuated IR (FLAIR) on the basis of the appearance of the pretreatment MRI [22, 23]. Peptide-specific immunological responses were analyzed by Enzyme-Linked ImmunoSpot (ELISPOT) assay (for details, see the Methods section in Additional file 3) [16, 24].

#### Molecular-genetic analysis

Chromosomal number aberrations (CNAs) were assessed by metaphase comparative genomic hybridization, as previously described [25]. Mutation of the isocitrate dehydrogenase (IDH)1 gene, and O6-methylguanine DNA methyltransferase (MGMT) promoter methylation, were also analyzed as previously described [16].

### Immunohistochemical analysis

Histopathological analyses were performed on 4-μm sections of formalin-fixed, paraffin-embedded tissues of paired pre- and post-vaccination obtained from Case 2. The expression of VEGF-A, VEGFR1, VEGFR2, CD34 (endothelial cell marker), PDGFR-β (pericyte marker), programmed cell death-1 (PD-1) and programmed cell death ligand-1 (PD-L1) (immune-checkpoint molecules), CD4 (helper T-cell marker), CD8 (CTL marker), Foxp3 (Treg marker), CD163 (tumor-associated macrophage [TAM] marker), nestin (neural stem/ progenitor cell marker), and cleaved caspase 3 (apoptosis marker) were analyzed [26, 27].

Immunofluorescence staining for VEGFR1, VEGFR2, and PDGFR-β expressions or VEGFR1, VEGFR2, and cleaved caspase 3, CD34, VEGFR1 and cleaved caspase 3, or Foxp3 and cleaved caspase 3 expressions was performed (for details, see the Methods section in Additional file 3).

### RNA extraction, cDNA synthesis, and quantitative real-time PCR

For quantitative real-time PCR (qPCR) of VEGF-A, VEGFR1, VEGFR2 and Foxp3, RNA was isolated from 10-μm sections of formalin-fixed, paraffin-embedded tissue using the “NucleoSpin total RNA FFPE XS” Kit (Macherey-Nagel) (for details, see the Methods section in Additional file 3) [28].

### Statistical analysis

PFS was defined as the date elapsed between treatment initiation and tumor progression. OS and PFS were analyzed based on the Kaplan-Meier test. All statistical analyses were performed with IBM SPSS statistics. Differences were considered to be statistically significant when *p* < 0.05.

## Results

### Patient characteristics

Four patients with primary glioblastoma were enrolled in this study (39–75 years old, two males and two females) between September 2014 and March 2018. All patients received 14 cycles of VEGFRs peptide vaccination (Table 2). All cases were IDH1-R132H wild-type and MGMT promoter methylation appeared in Case 2 and 4. 1,3-bis-Chloroethyl-1-Nitrosourea (BCNU) wafer was used in the surgical treatment of Case 2 and 3. The data of CGH analysis are summarized in Table 2.

**Table 2:** Patients’ Characteristics

Case	Age	PS	MIB-1 index	IDH1 Mutation	MGMT Methylation	CGH	Surgical removal	Radiation	TMZ cycles	Other treatment
1	75	1	40.1	wild	–	+ 7p15.2-qter, − 10, −15q, (−16qcen-13)	GTR	40Gy/15fr	22	–
2	39	1	11.1	wild	+	+1pter-34.1, −9pter-21, − 10q21.1-ter, +13q12.2–31, +17p12-q21.1, −18q23, −21q, +22qcen-13.1	GTR	60Gy/30fr	31	BCNU wafer
3	52	0	30	wild	–	-1pter-36.1, + 7, −9pter-21, − 10	GTR	60Gy/30fr	14	BCNU wafer
4	50	0	50	wild	+	+ 7, − 10, +12q15, +13q14.3–33	GTR	60Gy/30fr	14	–

*BCNU* bis-chloroethylnitrosourea, *CGH* comparative genomic hybridization, *GTR* gross total resection, *IDH* isocitrate dehydrogenase, *MGMT* O6 methylguanine DNA methyltransferase, *PS* performance status, *TMZ* temozolomide

### Adverse events

No major toxicity (grade 3 and 4) was found in this study. During this vaccination, Case 4 developed grade 1 local skin reaction at the injection sites with induration, redness, and swelling. No patients developed ulcers at the injection sites. No delayed wound healing or gastrointestinal bleeding were seen either. No other adverse events, such as arterial and venous thromboembolism, hypertension, and proteinuria, which were reported in the clinical study of bevacizumab, were detected.

### Clinical response

**Fig. 2:**
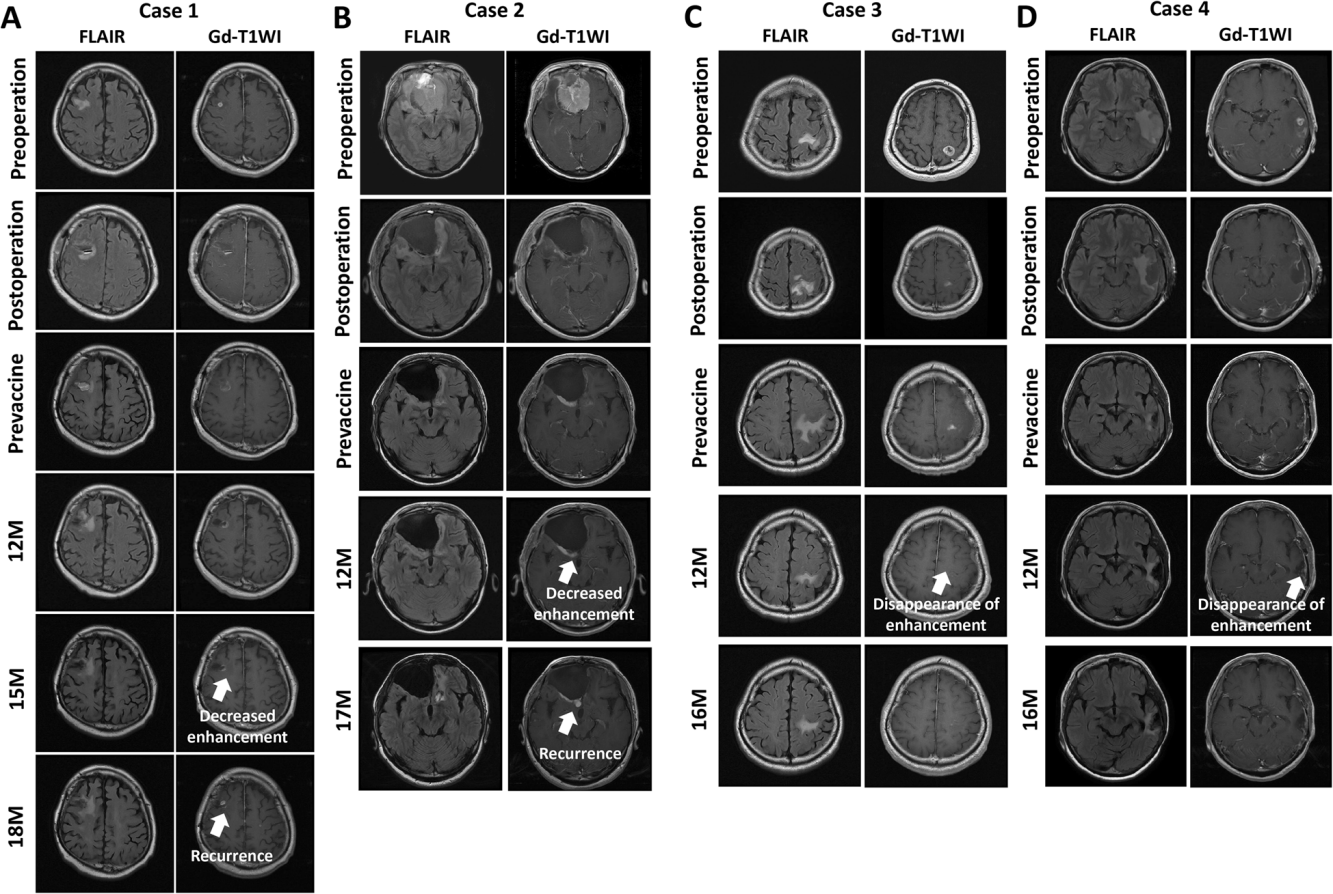
Radiographic images of enrolled patients. Radiographic images of Case 1(**a**), Case 2 (**b**), Case 3 (**c**), Case 4 (**d**). **a** The enhanced lesion was decreased 15 months, and recurrent lesion was observed at the removal site 18 months after the first vaccination. **b** The enhanced lesion was decreased 12 months, and recurrent lesion was observed at the removal site 17 months after the first vaccination. **c** The enhanced lesion disappeared 12 months after the first vaccination. **d** The enhancement lesion disappeared 12 months after the first vaccination

**Fig. 3:**
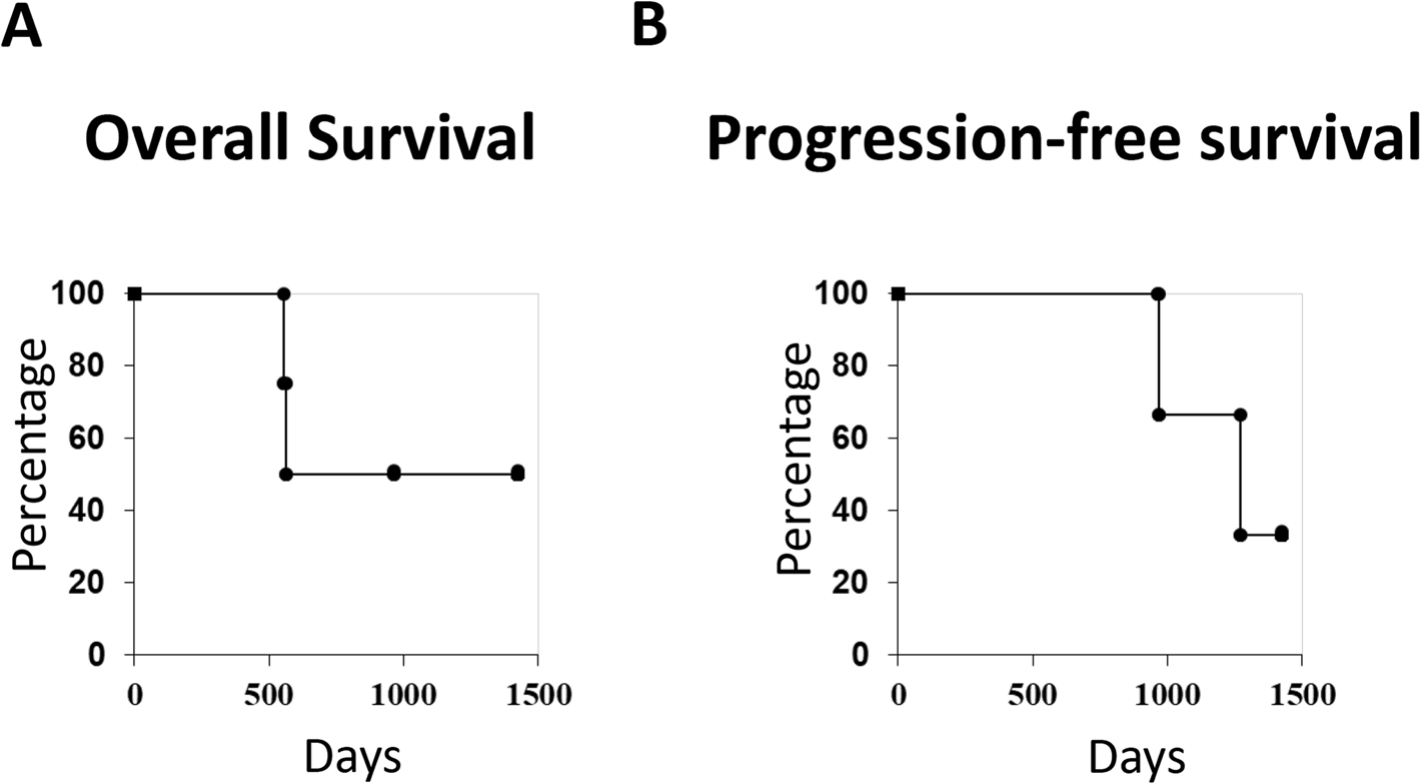
Clinical course of enrolled patients. **a** Overall survival of four patients. At the time of analysis, Case 3 and 4 still had CR. **b** Progression-free survival of four patients

Case 3 and 4 achieved complete response (CR). MGMT methylation was not detected in Case 3 (Fig. 2c, d). Although Gd-enhanced lesions were temporarily decreased in Case 1, and 2, Case 1 revealed progressive disease (PD) 10 months after the last vaccination and 9 months in Case 2 (Fig. 2a, b). In Case 2, the recurrent enhanced lesion was surgically removed again. The Kaplan–Meier curves for OS and PFS in four patients are shown in Fig. 3, respectively. At the time of analysis, Case 3 and 4 still showed CR (1425 and 962 days). Case 1 and 2 had already died (OS: Case 1, 967 days; Case 2, 1272 days).

### CD8+ T-cell response

In Case 3 and 4, CTLs specific for both VEGFR1 and 2 were induced after vaccination. Immunological monitoring could not be performed for Case 1 and 2, as the samples were lost because of a deep freezer fault (Table 3).

**Table 3:** Clinical results

Case No.	DTH	Vac cycles	Toxicity	ELISPOT (CTL)			CTL induction		PFS (days)	OS (days)	Evaluation after 12 M
				timing	R1	R2	R1	R2			
1	–	14	–	Before	N/A	N/A	N/A	N/A	554	967	PR
				After	N/A	N/A					
2	–	14	–	Before	N/A	N/A	N/A	N/A	562	1272	SD
				After	N/A	N/A					
3	–	14	–	Before	–	+	+	+	–	Still survive (1425)	CR
				After	+	3+					
4	+	14	–	Before	–	N/A	+	+	–	Still survive (962)	CR
				After	3+	2+					

*CTL* cytotoxic T lymphocyte, *DTH* delayed type hypersensitivity, *M* month, *N/A* not available, *OS* overall survival, *PD* progressive disease, *PFS* progression-free survival, *PR* partial response, *R* vascular endothelial growth factor receptor, *SD* stable disease, *Vac* vaccination

### Histopathological analysis

**Fig. 4:**
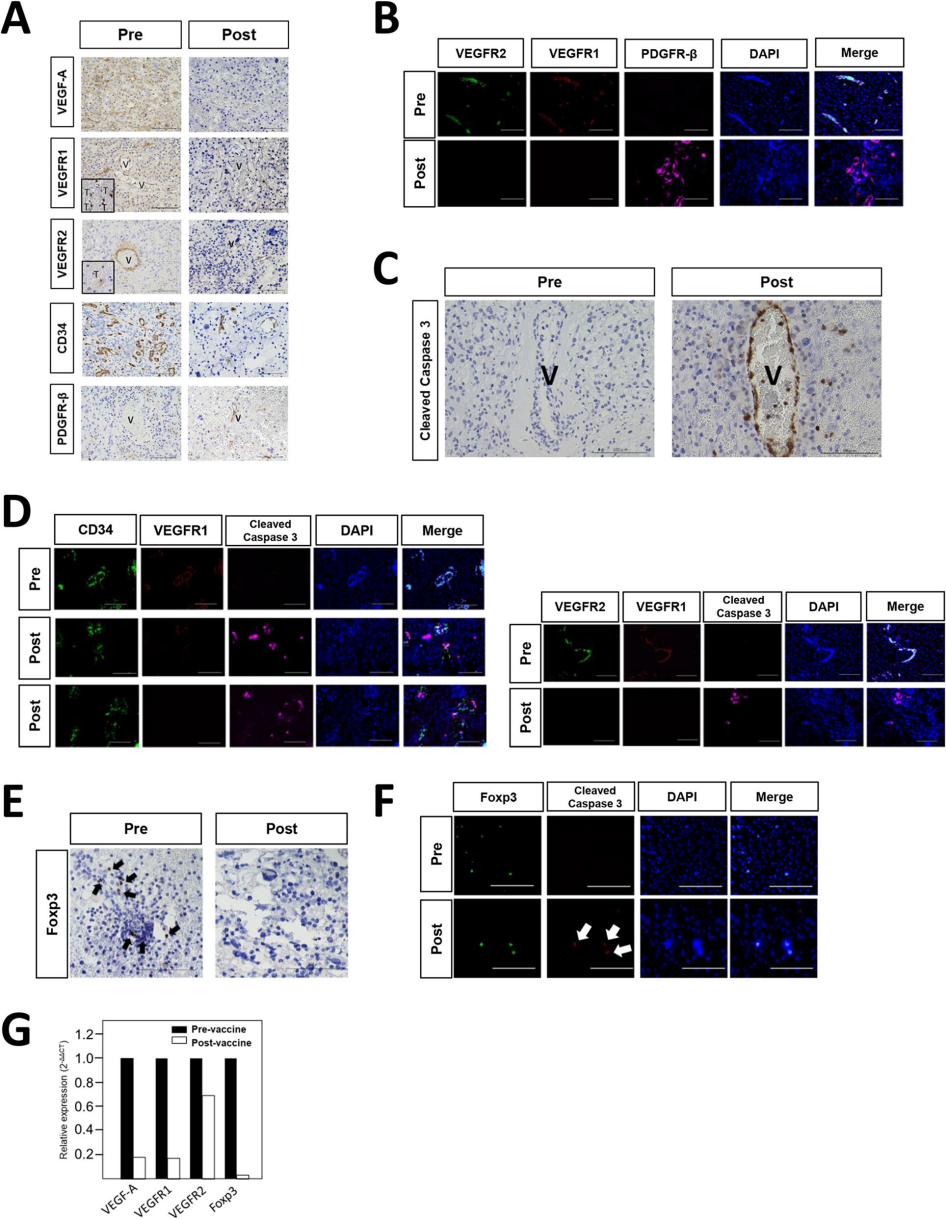
Histopathological images of the paired pre- and post-vaccination. **a** Histopathological analysis of VEGF-A, VEGFR1, VEGFR2, CD34 and PDGFR-β expressions in the tumor of pre- and post- vaccination. A square means tumor cells with positive VEGFR1 or VEGFR2 staining (T: tumor cell, V: vessel; original magnification, × 40; magnification bar, 100 μm). **b** Immunofluorescent analysis of VEGFR1, VEGFR2 and PDGFR-β expressions in tumor vessels in pre- and post- vaccination (original magnification, × 40; magnification bar, 100 μm). **c** Histopathological analysis of cleaved caspase3 expression on the tumor vessel of pre- and post- vaccination. (V: vessel; original magnification, × 40; magnification bar, 100 μm). **d** Immunofluorescent analysis of CD34, VEGFR1, and cleaved caspase3 expressions in the tumor of pre- and post- vaccination. Expression of cleaved caspase 3 was detected on the endothelial cells with slight VEGFR1 expression or without VEGFR1 expression (original magnification, × 40; magnification bar, 100 μm). **e** Histopathological analysis of Foxp3 expression in the tumor of pre- and post- vaccination (original magnification, × 40; magnification bar, 100 μm; black arrows, positive cells). **f** Immunofluorescent analysis of Foxp3 and cleaved caspase3 expressions in the tumor of pre- and post- vaccination (original magnification, × 40; magnification bar, 100 μm; white arrow, positive cells). **g** Relative gene expression of VEGF-A, VEGFR1, VEGFR2 and Foxp3 in the tumors of pre- and post- vaccination

The histological changes using pre- and post-vaccination glioblastoma specimens could be analyzed in Case 2. The analysis of endothelial cells stained by CD34 demonstrated that vessel diameter was smaller, and microvessel density (MVD) was lower after vaccination. Most tumor vessels exhibited strong VEGFRs expressions without PDGFR-β positive pericytes before vaccination. In contrast, tumor vessels after vaccination showed negative or slight expression of VEGFR1 and 2, and most endothelial cells were covered with PDGFR-β positive pericytes (Fig. 4a, b). Tumor cells with VEGFR1 or R2 expression were observed in the pre-vaccination tumor (Fig. 4a). VEGF-A expression after vaccination was also decreased compared with that before vaccination (Fig. 4a). There were fewer Foxp3-positive cells in the post-vaccination tumor compared with that of pre-vaccination (Fig. 4e). qPCR analysis revealed that the mean relative expressions of VEGF-A, VEGFRs, and Foxp3 genes in the post-vaccination tumors were lower than that of the pre-vaccination tumor (Fig. 4g). More number of cleaved caspase 3 positive cells were detected in post-vaccination tumor than in pre-vaccination tumor. In the post-vaccination tumor, the expression of cleaved caspase 3 was co-localized in endothelial cells with CD34-positive staining and Foxp3-positive cells (Fig. 4c, d, e, f). In contrast, the number of CD163, CD8, and CD4-positive cells did not change after vaccination. Immune checkpoint molecules, such as PD-1/PD-L1 and, a marker of glioma stem cell-like phenotype, such as nestin did not change either after vaccination (Additional file 1: Figure S1 A,B,C).

## Discussion

VEGF /VEGFRs signaling plays a pivotal role in the tumor angiogenesis and the development of the immunosuppressive tumor microenvironment in glioblastomas by inhibiting the maturation of dendritic cells (DCs) and stimulating the proliferation of Tregs, TAMs, and myeloid-derived suppressor cells (MDSCs) with VEGFRs expressions [7, 29–32]. Therefore, anti-angiogenic therapy targeting VEGF and/or VEGFRs, including VEGFRs peptide vaccination, has not only anti-angiogenic effects, but also immune-supportive effects [19, 26]. In addition, VEGFRs peptide vaccination has the advantages of immunotherapy. CTLs induced by the vaccination may persist in the long-term. In the present study, the synergistic activity of the combinational usage of VEGFRs peptide vaccination and TMZ-based chemotherapy was investigated for the patients with primary glioblastomas.

In this study, the disappearance of a radiographically enhanced lesion in the patient with unmethylated MGMT promoter was suggestive. The histopathological changes after the VEGFRs vaccination using paired pre- and post-vaccination specimens demonstrated that VEGFR1 and 2 peptide vaccination induced the normalization of vascular structure with decreased VEGFR1 and 2 expressions, and the reduction of MVD in the recurrent tumor after vaccination. We have previously reported the histopathological changes after the administration of bevacizumab (anti-VEGF-A monoclonal antibody) using actual human glioblastoma specimens resected in 3 different settings: glioblastomas before any treatment; glioblastomas resected following bevacizumab therapy; and recurrent glioblastomas after long-term bevacizumab therapy [26, 27, 33]. In these previous studies, the expressions of VEGFR1 and 2 were upregulated in recurrent glioblastomas after long-term bevacizumab therapy [33]. The present histopathological results might suggest that memory CTL induced by VEGFRs peptide vaccination may overcome the problems of anti-angiogenic molecular targeting agents, which include apparent drug resistance and rebound upregulated VEGF-A/VEGFRs signaling [34]. However, peptide-based vaccination results in the induction of T cell exhaustion. The transient upregulation of PD-1 during T-cell activation and its maintenance on chronically stimulated exhausted T cells enable PD-1 to negatively regulate T-cell function [35, 36]. Therefore, Immune checkpoint inhibition may exert synergic effects when administered with this type of CTL-mediated antitumor immunotherapy. In the future, we will reveal the difference in the target of inhibition, VEGF compared with VEGFRs using these valuable human tumor specimens.

Furthermore, the combinational usage of chemotherapy and immunotherapy was reported to be effective with synergistic activity [19–21]. The combination of TMZ and immunotherapy with fusions of dendritic cells (DCs) and glioma cells safely induced anti-tumor effects in patients with glioblastomas [37]. However, the Tregs population increases rapidly, known as the rebound phenomenon, during long-term TMZ therapy for glioblastomas [38]. Importantly, the present histopathological finding of cleaved caspase 3 also proved that VEGFR1 and 2 peptide vaccination could target wide variety of cells associated with tumor growth, such as vascular endothelial cells, tumor cells, and Foxp3 + Tregs expressing VEGFR1 and/or VEGFR2, which was considered as one of the rationales behind using VEGFRs peptide vaccination with TMZ. Previous study also demonstrated that VEGFR2-targeting treatment has the possibility of selectively killing Tregs, because Foxp3(+) Tregs express VEGFR2 [39, 40], which was compatible with the present results.

Although immunotherapy has become an increasingly available and vital cancer treatment option, it has some disadvantages. Immunotherapy’s potential side effects result from an overstimulated or misdirected immune response. However, fewer severe side effects were reported in the clinical setting. The clinical trials with peptide-based vaccine therapy using VEGFR-derived epitopes have been previously conducted for the patients with advanced gastrointestinal cancers and renal cell cancer, wherein the treatment exhibited the safety (Additional file 2: Table S1) [20, 41–47]. Chemotherapy attacks all rapidly-dividing cells within the body, effectively targeting fast-growing tumors. In contrast, immunotherapy takes longer time to work compared with other treatments [48].

The present study might demonstrate that the preliminary safety and immunogenicity of this approach. VEGFR1 and 2-specific CTLs inductions were detected under treatment with TMZ. In addition, paired pre- and post-vaccination specimens suggested that VEGFR1 and 2 peptide vaccination may possibly enhance the effects of TMZ. The limitation of the present study was the paucity of the number of enrolled patients. Further studies involving more patients are warranted to confirm the findings of this study.

## Conclusions

This is the first clinical trial of combinational therapy with VEGFRs peptide-based vaccines plus TMZ in patients with primary glioblastomas. Paired pre- and post-vaccination specimens suggested that VEGFRs peptide vaccination may possibly enhance the effects of TMZ. Further studies involving more patients are warranted to confirm the findings of this study.

## Supplementary information

**Additional file 1: Figure S1.** Other factors in the tumor microenvironment. The expression of PD-1 and PD-L1 (A), nestin (B), and CD 163, CD8, CD4 (C) in the tumors of pre- and post- vaccination. The number of positive cells did not change after vaccination. (original magnification, × 40; magnification bar, 100 μm; black arrows, positive cells).

**Additional file 2: Table S1.** Review of reported clinical trials using VEGFR1 or 2 peptide vaccine.

**Additional file 3.** Appendix.

## Data Availability

All data supporting the findings of this study are available within the article and its Supplementary Information Files and from the corresponding author on reasonable request.

## Abbreviations

BCNU: 1,3-bis-Chloroethyl-1-Nitrosourea
CAN: Chromosomal number aberrations
CR: Complete response
CT: Computed tomography
CTL: Cytotoxic T lymphocytes
DC: Dendritic cell
ELISPOT: Enzyme-linked immunoSpot
FLAIR: Fluid attenuated IR
Gd: Gadolinium
GMP: Good manufacturing practice
HPLC: High-performance liquid chromatography
IDH: Isocitrate dehydrogenase
MDSC: Myeloid-derived suppressor cells
MGMT: O6-methylguanine DNA methyltransferase
MRI: Magnetic resonance imaging
MVD: Microvessel density
OS: Overall survival
PDGFR: Platelet-derived growth factor receptor
qPCR: Quantitative real-time PCR
RT: Radiotherapy
TAM: Tumor-associated macrophage
TMZ: Temozolomide
VEGF: Vascular endothelial growth factor
VEGFR: Vascular endothelial growth factor receptor
