# Correction to: Clinical and histopathological analyses of VEGF receptors peptide vaccine in patients with primary glioblastoma - a case series

**DOI:** 10.1186/s12885-020-06783-8

**Published:** 2020-04-14

**Authors:** Ryota Tamura, Yukina Morimoto, Kenzo Kosugi, Mizuto Sato, Yumiko Oishi, Ryo Ueda, Ryogo Kikuchi, Hideaki Nagashima, Tetsuro Hikichi, Shinobu Noji, Yutaka Kawakami, Hikaru Sasaki, Kazunari Yoshida, Masahiro Toda

**Affiliations:** 1 Department of Neurosurgery, Keio University School of Medicine, 35 Shinanomachi, Shinjuku-ku, Tokyo, 160-8582 Japan; 2 Department of Neurosurgery, Hiratsuka City Hospital, Hiratsuka, Kanagawa 254-0019 Japan; 3 Division of Cellular Signaling Institute for Advanced Medical Research, Keio University School of Medicine, 35 Shinanomachi, Shinjuku-ku, Tokyo, 160-8582 Japan; 4 OncoTherapy Science, Inc., 3-2-1, Sakado, Takatsu-ku, Kawasaki City, Kanagawa 213-0012 Japan

**Correction to: BMC Cancer (2020) 20:196**

**https://doi.org/10.1186/s12885-020-6589-x**

Following publication of the original article [1], the authors reported an error in the author group.

It was reported that Tetsuro Hikichi was missing and the corrected author group is as follows:

Ryota Tamura^1^, Yukina Morimoto^1^, Kenzo Kosugi^1^, Mizuto Sato^1^, Yumiko Oishi^1^, Ryo Ueda^1^, Ryogo Kikuchi^2^, Hideaki Nagashima^1^, Tetsuro Hikichi^4^, Shinobu Noji^3^, Yutaka Kawakami^3^, Hikaru Sasaki^1^, Kazunari Yoshida^1^ and Masahiro Toda^1*^

The competing interests statement has been updated to:

TH is an employee of OncoTherapy Science, Inc.

The original article [1] has been corrected.

## References

[1] Tamura R, Morimoto Y, Kosugi K (2020). Clinical and histopathological analyses of VEGF receptors peptide vaccine in patients with primary glioblastoma - a case series. BMC Cancer.

