# Supplementary figures and images for: Clinical and histopathological analyses of VEGF receptors peptide vaccine in patients with primary glioblastoma - a case series
Source: BMC Cancer. 2020 Mar 12;20:196. doi: 10.1186/s12885-020-6589-x (PMC7066743; doi:10.1186/s12885-020-6589-x)

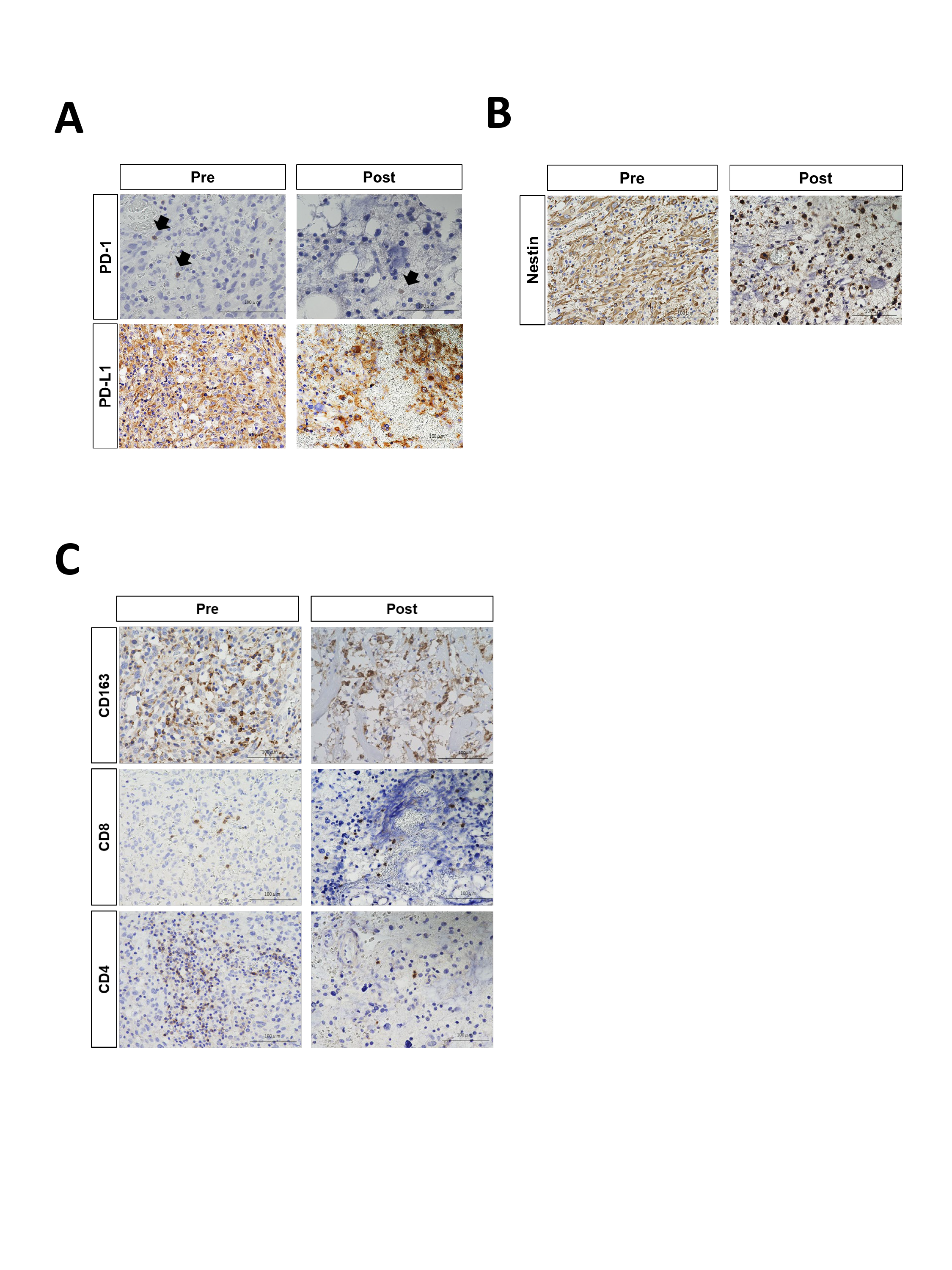

Supplement: Supplementary file 1 — Additional file 1: Figure S1. Other factors in the tumor microenvironment. The expression of PD-1 and PD-L1 (A), nestin (B), and CD 163, CD8, CD4 (C) in the tumors of pre- and post- vaccination. The number of positive cells did not change after vaccination. (original magnification, × 40; magnification bar, 100 μm; black arrows, positive cells). [file 12885_2020_6589_MOESM1_ESM.tif]
