# Supplementary material for: Clinical and histopathological analyses of VEGF receptors peptide vaccine in patients with primary glioblastoma - a case series
Source: BMC Cancer. 2020 Mar 12;20:196. doi: 10.1186/s12885-020-6589-x (PMC7066743; doi:10.1186/s12885-020-6589-x)
Supplement: Supplementary file 2 — Additional file 2: Table S1. Review of reported clinical trials using VEGFR1 or 2 peptide vaccine. [file 12885_2020_6589_MOESM2_ESM.docx]

| **Author, year** | **Phase** | **Disease** | **Number** | **HLA** | **Peptide** | **Dose (/body)** | **Combinational**  **chemotherapy** | **CTL induction** | **Toxicity** |
| --- | --- | --- | --- | --- | --- | --- | --- | --- | --- |
| **MiyazawaM. 2010 (20)** | I | pancreas cancer | 18 | 24:02 | VEGFR2 （OTS-102） | 0.5mg, 1mg  2mg | gemcitabine | 0.5mg:3/6 (50%) 1.0mg:4/6 (67%) 2.0mg:4/6 (67%) | Grade 4:0 Grade 3:1 (duodenal hemorrhage), 2(AST and ALT↑), 2(leukopenia) |
| **Masuzawa T. 2012 (45)** | I/II | gastric cancer | 22 | 24:02 | VEGFR1 and 2（OCV-101・OTS-102） | 1mg | S-1＋cisplatin | VEGFR1:18/22 VEGFR2:18/22 | Grade 3 or 4: leukopenia and anemia |
| **Iinuma H.2014 (43)** | I | esophageal cancer | 11 | 24:02 | VEGFR1 and 2（OCV-101・OTS-102） TTK protein kinase (TTK) up-regulated lung cancer10(URLC10) insulin-like growth factor-ⅡmRNA binding protein 3 (KOC1) | 0.5mg 1.0mg 3.0mg | 5-FU＋cisplatin | All patients showed CTL responses at least one of 5 kinds of peptide antigens | Grade 4: 0 Grade 3: leukopenia, anemia, |
| **Hazama S.2014 (41)** | I | colorectal cancer | 19 | 24:02 | VEGFR1 and 2（OCV-101・OTS-102） RNF43 TOMM34 KOCI | 0.5mg 1.0mg 3.0mg | ― | VEGFR1 0.5mg:1/3 1.0mg:1/3 3.0mg:5/6 VEGFR2 0.5mg:2/3 1.0mg:2/3 3.0mg:3/6 | Grade 3 or 4: 0 |
| **Yoshimura K.2013 (47)** | I | metastatic kidney cancer | 18 | 24:02 02:01 | VEGFR1（OCV-101・MPS-400） | 0.5mg 1.0mg 3.0mg |  | VEGFR1 0.5mg:5/6 1.0mg:5/6 3.0mg:3/6 | Grade 3 or 4: 0 |
| **Hazama S.2014 (42)** | II | colorectal cancer | 96 | 24:02 | VEGFR1 and 2（OCV-101・OTS-102） RNF43 TOMM34 KOCI | 3mg | mFOLFOX6,XELOX | － | Grade 4: 5 (interstitial pneumonia,Neutropenia) 1:embolism |
| **Shibao S. 2017 (16)** | I/II | Recurrent glioblastoma | 8 | 24:02 | VEGFR1 and 2（OCV-101・OTS-102） | 2mg | ― | VEGFR1:7/8 VEGFR2:1/8 | Grade 3: 0 |
| **Kikuchi R. 2019 (17)** | I/II | Recurrent glioblastoma | 10 | 24:02 | VEGFR1 and 2（OCV-101・OTS-102）,  LY6K, DEPDC1,  KIF20A, and FOXM1 | 2mg | ― | VEGFR1:3/6  VEGFR2:2/6 | Grade 1: 1(skin flare)  Grade 2: 1(wound infection), 1 (herpes zoster) |
| **Tamura R. 2019 (18)** | I/II | NF2 | 7 | 24:02  02:01 | VEGFR1 and 2（OCV-101・OTS-102, MPS-400・MPS-464） | 2mg | ― | VEGFR1:7/7  VEGFR2:6/7 | Grade 1: 5 (local skin reaction), 1 (hypertension)  Grade 2: 4 (neutropenia)  Grade 3: 1 (diverticulitis)  Grade 5: 1 (intracranial hemorrhage)* |

**Supplementary Table 1: Review of reported clinical trials using VEGFR1 or 2 peptide vaccine**

CTL, cytotoxic T lymphocyte; NF2, neurofibromatosis type 2; VEGFR, vascular endothelial growth factor receptor

* The aforementioned Independent Data Monitoring Committee concluded that the experimental vaccine was not likely to be related to this adverse event.
