# Supplementary material for: Clinical and histopathological analyses of VEGF receptors peptide vaccine in patients with primary glioblastoma - a case series
Source: BMC Cancer. 2020 Mar 12;20:196. doi: 10.1186/s12885-020-6589-x (PMC7066743; doi:10.1186/s12885-020-6589-x)
Supplement: Supplementary file 3 — Additional file 3. Appendix. [file 12885_2020_6589_MOESM3_ESM.docx]

**Supplementary Appendix**

**This appendix has been compiled by the authors to provide readers additional information about their work.**

**Supplement to:**

**Tamura R, et al. Clinical and Histopathological analysis of VEGF receptors peptide vaccine in patients with primary glioblastoma - a case series**

**Supplementary Methods**

*Isolation and stock of peripheral blood mononuclear cells*

Peripheral blood cells were obtained from patients within 2 weeks before the first cycle, after 8, 12 and 14 cycles (3, 6, and 12 months). Peripheral blood mononuclear cells (PBMCs) were isolated immediately by Ficoll-Paque Plus density gradient solution (GE Healthcare Little Chalfont, UK), suspended in Cell Banker (ZENOAC, Fukushima), and frozen and stored at -80℃ in deep freezer.

*ELISPOT assay and CD8+ T-cell responses to peptide stimulation.*

ELISPOT assay was performed to evaluate the specific CD8 positive T cell response by contract research organization “OncoTherapy Science, Inc.” and “Cancer Precision Medicine, Inc.” [1], as described in our previous paper [2]. The positivity for an antigen-specific CD8+ T-cell response was quantitatively defined according to a modified evaluation tree algorithm based on that described previously [3]. In brief, the number of peptide-specific spots was calculated as the average of triplicates by subtracting the number of spots in the control well from that observed in the well with peptide-pulsed stimulator. Positivity for each antigen-specific CD8+ T-cell response was classified into four grades (−, +, ++, and +++) depending on the number and variability of peptide-specific spots at different responder/stimulator ratios. Induction of CTLs due to vaccine was judged when the positivity increased after vaccine.

Molecular-genetic analysis

Chromosomal number aberrations (CNAs) were assessed by metaphase comparative genomic hybridization, as described previously. Briefly, crude tumor DNA from formalin-fixed paraffin-embedded (FFPE) tissue was amplified by degenerate oligonucleotide primed-polymerase chain reaction (DOP-PCR), and labeled with another DOP-PCR, using digoxigenin (DIG)-11-dUTP (Roche, Mannheim, Germany). The reference DNA was amplified from 50 ng of DNA from a healthy man or woman, and labeled with biotin-dUTP (Roche). The probe mixture was denatured and hybridized to normal metaphase spreads (Vysis, Downers Grove, IL, USA). Unhybridized probes were washed out, and the metaphase spread was incubated with a fluorescein isothiocyanate-conjugated anti-DIG antibody (Roche) and rhodamine-conjugated avidin (Roche). Preparations were washed and counterstained with 4,6-diamino-2phenylinodole in antifade solution. Red, green, and blue images were acquired, and ratios of fluorescence intensity along chromosomes were quantitated using the CytoVision® Analysis System (Applied Imaging, San Jose, CA, USA) [4,5].

Mutation of the IDH1 gene was assessed as follows: FFPE tissue sections were examined for isocitrate dehydrogenase 1 (IDH1)-R132H mutation using immunohistochemistry, with an anti-mutant IDH1 antibody (1:20, H09, Dianova) [6]. O6-methylguanine DNA methyltransferase (MGMT) promoter methylation was evaluated using anti-MGMT antibody (1:100, MT3.1, Merck Millipore).

*Immunohistochemical analysis*

Histopathological analyses were performed on 4-µm sections of formalin-fixed, paraffin-embedded tissue of paired pre- and post-vaccine obtained from Case 2. Immunohistochemistry was performed with anti-VEGF-A antibody (1:200, JH121, Merick Millipore), anti-VEGFR-1 antibody (1:100, AF321, R&D Systems), anti-VEGFR-2 antibody (1:600, 55B11, Cell Signaling), anti-CD34 antibody (1:100, QBEnd 10; Dako), anti-PDGFR-β antibody (1:50, Y92; Abcam), anti-PD-1 antibody (1:50, NAT105, Abcam), anti-PD-L1 antibody (1:500, 28-8, Abcam), anti-CD4 antibody (1:200, 1F6, Nichirei Biosciences Inc.), anti-CD8 antibody (1:100, 144B, Abcam), anti-Foxp3 antibody (1:100, ab54501, Abcam), and anti-CD163 antibody (1:100, ab87099, Abcam), anti-nestin antibody (1:100, 10C2, Chemicon), and anti-cleaved caspase 3 antibody (1:200, ASP 175, CST). The antigen retrieval was performed in citrate buffer (pH 6 for VEGFR1, PD-1, CD4, Foxp3, CD163 and Nestin), or in Tris buffer (pH 9 for VEGF-A, VEGFR2, CD34, PDGFR-β, PD-L1, CD8, cleaved caspase 3) using microwave irradiation or autocrave (PD-L1), and the products were visualized with peroxidase-diaminobenzidine reaction. All expressions were assessed by consensus of four authors with blinded clinical information (RT, MS, and YM).

*Immunofluorescent analysis*

Immunofluorescence staining for VEGFR1, VEGFR2, and PDGFR-β expressions or VEGFR1, VEGFR2, and cleaved caspase 3, CD34, VEGFR1 and cleaved caspase 3, or Foxp3 and cleaved caspase 3 expressions was performed to evaluate the vascular characteristics. Tissue sections were incubated with antibodies against VEGFR1 (1:100, AF321, goat polyclonal IgG; R&D Systems), VEGFR2(1:100, EIC, mouse monoclonal IgG; Abcam), PDGFR-β (1:50, Y92, rabbit monoclonal IgG; Abcam), CD34 (1:200, mouse monoclocal IgG; QBEnd 10; Dako), cleaved caspase 3 (1:200, rabbit polyclonal IgG; ASP 175, CST) or Foxp3 (1:100, mouse monoclonal antibody, ab54501, Abcam) overnight at 4˚C. Appropriate secondary antibodies, Alexa Fluor 488 donkey anti-mouse IgG, Alexa Fluor 568 donkey anti-goat IgG, or Alexa Fluor 647 donkey anti-rabbit IgG were used(1:200). Finally, the sections were mounted with mounting medium for ﬂuorescence with 4′,6′-diamidino-2-phenylindole (DAPI; Santa Cruz Biotechnology). Cell images were captured by ﬂuorescencemicroscopy (Biorevo BZ-9000, KEYENCE).

*RNA extraction, cDNA synthesis, and quantitative real-time PCR*

For quantitative real-time PCR (qPCR), RNA was isolated from 10-μm sections of formalin-fixed, paraffin-embedded tissue using the “NucleoSpin total　RNA FFPE XS” Kit (Macherey-Nagel). cDNA was subsequently obtained using SuperScript II First Strand Synthesis System with random hexamers (Invitrogen). qPCR was performed by using 10 μl SYBR Green reagent (Applied Biosystems, USA), 2 μl synthesized cDNA , 2 μl of the same primers that were used in standard PCR and 6 μl of molecular grade water in a total volume of 20 μl. qPCR was run in triplicates on ABI StepOnePlus real time PCR machine (Applied Biosystem, Foster, USA). Amplification was performed under the following conditions: 10 minutes at 95˚C, 55 cycles of 95˚C for 15 seconds and 60˚C for 60 seconds. All experiments included negative controls (nontemplate water instead of cDNA). The qPCR data were analyzed using the comparative CT method. Briefly, the difference in cycle threshold, ΔCT, was determined as the difference between the tested gene and human GAPDH. Data were then normalized to GAPDH cDNA. The fold change was calculated as 2^-ΔΔCT^[7,8].

Primer sequences are listed as the following:

VEGF-A forward (fwd): GAGATGAGCTTCCTACAGCAC

VEGF-A reverse (rev): TCACCGCCTCGGCTTGTCACAT

VEGFR1 fwd: CAGGCCCAGTTTCTGCCATT

VEGFR1 rev: TTCCAGCTCAGCGTGGTCGTA

VEGF2 fwd: CCAGCAAAAGCAGGGAGTCTGT

VEGFR2 rev: TGTCTGTGTCATCGGAGTGATATCC

Foxp3 fwd: GGCCCTTCTCCAGGACAGA

Foxp3 rev: GCTGATCATGGCTGGGTTGT

GAPDH fwd:TGAACGGGAAGCTCACTGG

GAPDH rev:TCCACCACCCTGTTGCTGTA

**Supplementary References**

1.Ranieri E, Popescu I, Gigante M. CTL ELISPOT assay. Methods Mol Biol.2014;1186:75- 86.

2.Shibao S, Ueda R, Saito K, Kikuchi R, Nagashima H, Kojima A, Kagami H, Pareira ES, Sasaki H, Noji S, Kawakami Y, Yoshida K, Toda M. A pilot study of peptide vaccines for VEGF receptor 1 and 2 in patients with recurrent/progressive high grade glioma. Oncotarget.2018;9:21569- 21579.

3. Kono K, Iinuma H, Akutsu Y, Tanaka H, Hayashi N, Uchikado Y, Noguchi T, Fujii H, Okinaka K, Fukushima R, Matsubara H, Ohira M, Baba H, Natsugoe S, Kitano S, Takeda K, Yoshida K, Tsunoda T, Nakamura Y. Multicenter, phase II clinical trial of cancer vaccination for advanced esophageal cancer with three peptides derived from novel cancer-testis antigens. J Transl Med. 2012;10:141.

4.Hirose Y, Aldape K, Takahashi M, Berger MS, Feuerstein BG. Tissue microdissection and degenerate oligonucleotide primed-polymerase chain reaction (DOP-PCR) is an effective method to analyze genetic aberrations in invasive tumors. J Mol Diagn.2001;3:62–67.

5.Miwa T, Hirose Y, Sasaki H, Ezaki T, Yoshida K, Kawase T. Single-copy gain of chromosome 1q is a negative prognostic marker in pediatric nonependymal, nonpilocytic gliomas. Neurosurgery.2011;68:206-212.

6.Capper D, Zentgraf H, Balss J, Hartmann C, von Deimling A. Monoclonal antibody specific for IDH1 R132H mutation. Acta Neuropathol.2009;118:599–601.

7.Boeckx C, Wouters A, Pauwels B, Deschoolmeester V, Specenier P, Lukaszuk K, Vermorken JB, Pauwels P, Peeters M, Lardon F, Baay MF. Expression analysis on archival material: comparison of 5 commercially available RNA isolation kits for FFPE material. Diagn Mol Pathol.2011;20:203-211.

8.Tamura R, Ohara K, Morimoto Y, Kosugi K, Oishi Y, Sato M, Yoshida K, Toda M. PITX2 Expression in Non-functional Pituitary Neuroendocrine Tumor with Cavernous Sinus Invasion. Endocr Pathol.2019;30:81-89.
